# Supplementary material for: Are carbon emissions trading and green financial instruments synergistic? -Comprehensive quantitative research based on content analysis
Source: PLoS One. 2024 Mar 7;19(3):e0298601. doi: 10.1371/journal.pone.0298601 (PMC10919601; doi:10.1371/journal.pone.0298601)
Supplement: S1 Data — (ZIP) [file pone.0298601.s001.zip › Data/地级市Z_F工作报告.zip!/╣π╬≈/║╪╓▌╩╨/└┤╫╘╣1⁄2╓┌║┼í╛┬φ┐╦╩2╛▌═°í┐_2015.docx]

各位代表：

现在，我代表市人民政府向大会作政府工作报告，请予审议，并请市政协委员和列席会议的同志提出意见。

一、2014年工作回顾

过去一年，面对复杂严峻的宏观形势和繁重的改革发展任务，全市人民在自治区党委、政府和市委的坚强领导下，沉着应对、积极作为、精准发力，统筹推进稳增长、促改革、调结构、惠民生各项工作，经济社会发展保持良好势头，实现了稳中提质、稳中增效、稳中向好。初步统计，全市实现地区生产总值440亿元，增长6.0%，一、二、三产增加值分别增长5.0%、5.5%和6.7%；财政收入40.6亿元，增长13.5%；固定资产投资524亿元，增长16.0%；规模以上工业增加值增长6.5%；城镇居民人均可支配收入增长11.2%；农民人均纯收入增长13.0%；城镇登记失业率2.91%，居民消费价格指数1.9%。一些事关贺州当前和长远发展的重大事项捷报频传。

——科学发展思路取得大突破。按照自治区党委彭清华书记提出“贺州要发挥向东开放排头兵作用，建成广西对接东部和中部地区的重要门户和枢纽”的指示精神，全面启动实施“向东发展战略”，坚持特色农业、特色旅游、特色城镇“三管齐下”，全力打造宜居宜商的美丽贺州，科学发展定位更加清晰、明确。

——城市规划编制取得大突破。抢抓贺州成为广西唯一的全国“多规合一”试点市县的历史机遇，全面启动 “多规合一”编制工作。同时，启动了“十三五”规划、城市总规、“生态新城” “一江两岸” 概念性总规及核心区控制性详规、园区规划和17个重点镇规划编制和修编工作，对城市发展空间的管控力明显提升，未来发展蓝图更加美好。

——交通优先发展取得大突破。贵广高铁建成通车，并开通运营贺州直达南宁高铁动车，将贺州至广州、至南宁车程分别缩至1个半小时和3个半小时左右，贺州昂首迈进了“高铁时代”，加快融入“高铁经济带”，时空格局得到极大优化。同时，永贺高速公路实现复工，贺州支线机场、通用机场、警用和应急救援机场项目前期工作正加速推进。

——重大项目投资取得大突破。全市投资增速首次赶上全区水平，市级层面重大工业项目新开工数、完成投资额分别是上年的2.3倍和1.5倍，工业投资和产业投资的比重明显提升。特别是，提前谋划了总投资达6000多亿的657个“十三五”重大项目，为我市后续发展夯实了基础。

——项目融资工作取得大突破。全市金融机构贷款余额279.6亿元，增长15.6%。争取政策性融资超100亿元，获国开行贷款授信72.34亿元，支持棚户区改造工程建设；市城投公司成功发行10亿元公司债用于基础设施建设；完成亚行贷款、德国促进贷款等一批项目的计划申报，为我市重大项目投资后续增长提供了有力支撑。

——“两个千亿元产业”取得大突破。碳酸钙和新型建筑材料“两个千亿元产业”乘势而上，其中碳酸钙产业规上企业完成总产值49.33亿元，增长23.4%，占规上工业总产值比重14.5%。中国冶金地质总局投资40亿元的碳酸钙产业项目、广西旭腾实业集团投资19.6亿元的新型建筑材料纳米科技板项目落户贺州，加快带动了“两个千亿元产业”集聚发展。

——生态环境建设取得大突破。贺江、桂江、龟石水库水质进一步改善，城区空气优良率达98.4%。贺州市生态健康产业发展规划已获自治区批复实施。富川瑶族自治县列入“国家生态文明先行示范区”，昭平县列入“全国生态文明示范工程试点县”；姑婆山景区成为广西唯一的全国生态旅游示范区；昭平七冲、大桂山鳄蜥两个自然保护区成为国家级自然保护区。

——民生重点领域取得大突破。在全国率先整合资源推进新型农村社区建设，完成280个农村幸福院、49个五保村和60个新型农村社区建设，昭平镇城东社区、富阳镇铁耕社区入选“全国综合减灾示范社区”。贺州高中、市实验中学如期搬迁新址办学，龙山小学、八步三中、平桂三中、平桂一幼等新建学校竣工投入使用，经多年努力结束了建市以来未新增一所城区中小学校的历史，有效解决了城区义务教育“大班额”问题。

一年来，我们主要做了以下工作：

（一）深化改革开放，经济发展活力持续增强。大胆探索建立跨省区产业合作新模式，加快粤桂县域经济产业合作示范区合作共建；稳步实施桂贺一体化发展规划，推进区域合作互动和发展优势互补。积极稳妥开展扩权强镇工作，抓好17个重点镇城乡一体化示范工程，有效带动全市新型城镇化发展。完成贺达纸业、市冶炼总厂的重组改制工作，组建了广西贺州正赢发展集团、正润发展集团。启动创新驱动发展战略，实施“引智入贺”工程，聘请了一批国内教育界专家和市政府经济发展、规划顾问，完善了重大决策专家咨询论证制度。优化行政审批流程，市级行政审批事项办理时限平均压缩了3.95个工作日，办结提速19.7%；市本级新开业企业户数增长15.3%，新增注册资本增长336.7%。同时，扎实推进区域合作交流，积极开展了重点产业招商，与世界500强企业日本轻金属株式会社合作共建的偏析法高纯铝项目顺利开工，与法国欧尚集团签订了共建城市商业综合体协议。全年新签招商引资项目399个，合同投资总额318.95亿元，增长15.9%；招商引资项目（含续建）到位资金301.67亿元。

（二）夯实发展支撑，基础设施建设上新台阶。启动实施综合交通基础设施大会战，贺州至富川一级公路（贺街至广贺高速公路贺州东出口段）、富裕至马江二级公路、三合至马江二级公路项目实现开工；国道323线改造、昭黄二级路绕城线（含马滩大桥）、八步南乡经大汤至广东福堂二级公路等项目加快建设；洛湛铁路扩能改造、贺州至来宾高速公路、贺江扩能工程、桂江航道工程等一批重大交通项目前期工作稳步推进。能源供给保障加强，启动了华润电厂二期、垃圾焚烧发电等重大能源项目前期工作；规划建设水电、风电、光伏等一批可再生能源项目；抓好县县通天然气工程项目建设，建成苍梧—贺州天然气管道、贺州市天然气专供管道等一批天然气项目；推动我市电力用户和发电企业直接交易试点工作。水利建设稳步推进，贺江整治工程进展顺利，江北东路堤已开工建设；综合治理河段总长34公里，完成病险水库除险加固主体工程33座；新增和改善灌溉面积37.2万亩，路花水库工程（贺州市备用水源）前期工作基本完成。年内解决了15.78万农村人口饮水不安全问题。

（三）推进结构调整，特色产业发展步伐加快。在碳酸钙产业蓬勃发展的同时，加快培育壮大战略性新兴产业，新材料、食品药品产业总产值分别增长26.2%、21.3%，占规上工业企业总产值比重分别为7.9%、11.4%。土地卫片执法监督检查成绩全区排名第一，建设高标准基本农田12万亩，粮食生产“十一连增”；富川现代特色果蔬产业（核心）示范区成功通过验收；我市成为全国唯一实现国家级出口食品农产品质量安全示范区全覆盖的地级市；与香港渔农署合作启动供港蔬菜“直通车” 项目，出口备案基地面积达25.8万亩。黄姚古镇、姑婆山景区创5A工作加速推进，十八水景区、大桂山国家森林公园成为广西生态旅游示范区。全市接待游客总人数增长19.3%，旅游总收入增长27.2%。

（四）突出生态宜居，“美丽贺州”建设深入推进。持续强化区域生态环境恢复和保护，深入实施“绿满八桂”工程，完成造林面积29.5万亩，义务植树651万株，全市森林覆盖率达72.91%。城区生活垃圾无害化处理率、污水处理率分别达100%和80%。提前完成“十二五”淘汰落后产能水泥、铁合金、造纸的工作目标任务。完成 “美丽贺州·清洁乡村”阶段活动，农村环境综合整治取得实效。通过“广西园林城市”验收，荣获第九届“南珠杯”竞赛优秀奖。“一江两岸”建设开发、市容市貌综合提质三年行动启动实施，稳步推进旧城区排水管网改造、担杆岭水厂二期工程等项目建设，全市城镇化水平达43%，城市建成区人口升至34万人，建成区面积扩展到38平方公里。

（五）注重改善民生，人民群众生活持续向好。筹资33.6亿元实施的10大项48小项为民办实事工程，除土地整理项目正在有序推进外，其余均已完成。城镇新增就业、就业困难人员再就业、农村劳动力转移就业等工作扎实推进。新增棚户区改造开工建设4913套，保障性安居工程开工建设4400多套，实施农村危房改造工程1.28万户。实施农村居民住房政策性保险项目，惠及43万户农民；城乡低保、五保供养等社会救助惠及群众33.78万人。发明专利申请516件，指标完成率全区排名第三；荣获第四届广西发明创造成果展览交易会金奖2项、银奖3项；桂东电子公司1项发明专利荣获“中国专利优秀奖”。组建了贺州职业学院，贺州学院与凤凰卫视集团合作建设“数字媒体产业人才项目实训基地”。《莲花雨》获全国曲艺最高奖“牡丹奖”提名奖。完成广播电视村村通、村级公共服务中心等项目年度建设任务。八步贺街镇成为中国历史文化名镇，富川福溪村成为中国历史文化名村，昭平新华村列入第三批中国传统村落。贺州籍运动员蒋惠花勇夺南京青奥会首金。贺州市人民医院与广州市第一人民医院建立对口支援关系；全市在建卫生基建项目91个，总建筑面积64万平方米，总投资近33亿元，增加病床2600张。有序实施单独两孩政策，低生育水平持续稳定。顺利完成食品药品监管体制改革。稳步推进扶贫开发和革命老区扶贫攻坚“三年帮扶行动”，减少贫困人口8.17万人。强化信访问题源头预防，安全生产、普法教育、水库移民安置、“打黄扫非”行动等工作进一步加强，“打盗抢”工作全区排名第一。

（六）持续转变作风，政府建设呈现新气象。深入开展群众路线教育实践活动，严格落实中央“八项规定”，纠正“四风”。市政府印发文件和召开全市性会议分别精减32%、21%。全市公共财政安排的“三公”经费下降18.1%。基本完成市县政府机构改革。完善政府内部审计、监察制度，开展清退干部职工违规多占住房等专项整治。自觉接受人大法律监督和工作监督，主动接受政协民主监督，人大代表议案建议和政协提案办结率100%，密切联系各民主党派、工商联、人民团体和社会各界人士。

一年来，驻梧管理、编制、外侨、统计、税务、史志、档案、民族宗教、机关事务、住房公积金、农机、供销、二轻联社、地震、气象、水文、消防、邮政、通信等各项工作取得新进展，工会、共青团、妇女儿童等事业取得新进步，人防、民兵预备役和国防动员、国防教育深入开展，双拥共建工作取得新成绩。

各位代表，一年来所取得的成绩，是自治区党委、政府亲切关怀、正确领导的结果，是市委总揽全局、科学决策的结果，是市人大依法监督和市政协民主监督的结果，是全市各族人民团结拼搏、开拓进取的结果。在此，我代表市人民政府，向全市广大干部群众，向全体人大代表和政协委员，向各民主党派、工商联、各人民团体和社会各界人士，向驻贺部队和武警官兵，向关心支持贺州发展的老领导、老同志，表示衷心感谢并致以崇高敬意！

过去的一年，经济下行压力不断加大，我市产业结构不尽合理、传统资源型产业比重过高、统筹环境保护和转型发展难等问题凸显，企业融资难、用地难，工业电价高，受贺江水污染整治和市场制约的影响，一批企业停产减产，个别大型企业未能达产达效，导致全市GDP增速、规上工业增加值增速两大指标低于预期目标。同时，我市经济社会发展中还存在民生基础设施欠账多、历史遗留问题多、城市管理水平不高、政府部门执行力亟需增强等问题。对此，我们一定高度重视，采取更有力措施，化解难题，全力推进我市经济社会平稳健康发展。

二、2015年工作安排

2015年是“十二五”收官之年，也是全面落实党的十八届四中全会精神的开局之年，做好今年工作意义重大。当前，国家经济发展进入新常态，发展的速度、方式、结构、动力、机制正发生重大转变，我市既面临新常态的趋势性变化，又有后发展欠发达地区的差异性特征，固有的矛盾更加突出，新问题、新情况、新挑战也不断出现，经济持续下行的态势和发展的环境仍然非常严峻。但总体来看，我市仍然处于大有可为的战略机遇期。全国“多规合一”试点工作花落贺州，为我市提供了建市以来最大的政策红利；桂粤黔三省区联手打造“贵广高铁经济带”，我市地处中国—东盟自由贸易区、西部大开发和泛珠三角区域合作的战略结合点，作为珠三角经贵广高铁进入大西南第一站的区位优势更加突显，将更有效的把矿产、生态等资源优势转化为强大的产业发展动力。对此，我们既要增强忧患意识，充分估计面临的困难和挑战，把干事创业谋发展作为最大责任；又要善于抓住机遇，主动作为，开拓进取，努力提高新常态下推动发展的能力和水平。

今年政府工作的总体要求是：全面贯彻落实党的十八大、十八届三中、四中全会和中央、全区经济工作会议以及习近平总书记系列重要讲话精神，主动适应经济发展新常态，坚持稳中求进工作总基调，牢牢把握建设“贵广高铁经济带”等重大机遇，以扩量提质增效为中心，以实施“多规合一”为切入点，以壮大“两个千亿元产业”为龙头，构建特色优势支柱产业体系和特色城镇体系，突出驱动创新，完善基础设施，加强生态建设，深化改革开放，切实保障和改善民生，加快建设法治政府，促进经济平稳健康发展与社会和谐稳定。

今年我市经济社会发展的主要预期目标为：地区生产总值增长9%；财政收入增长10%；规模以上工业增加值增长15%；全社会固定资产投资增长16%；社会消费品零售总额增长10%；居民消费价格指数增幅控制在3%以内，外贸进出口总额增长10%；城镇居民人均可支配收入增长 10%；农民人均纯收入增长10%；城镇化率提高到45%，城镇新增就业人数1.2万人，节能减排指标控制在自治区下达的目标范围内。

确定上述目标，充分考虑了适应新常态、实现“十二五”既定目标、发展要素支撑等综合因素。在经济新常态的大背景下，我市既要保持合理较快的发展速度，更要注重提高质量和效益；既要采取有针对性的措施稳增长，又要做好打基础利长远强后劲的工作。我们要树立新的理念，谋划新的举措，实实在在提升经济社会发展的整体素质、整体水平和整体竞争力。为此，今年重点抓好以下工作：

（一）狠抓“多规合一”，统领区域协调发展。坚持“先行先试、可以突破”原则，建立一个市县“一本规划、一张蓝图”的规划衔接协调机制，探索新常态下经济社会一体化发展的新路径。

突出规划引领作用。将“多规合一”试点与“十三五”规划有机结合，科学编制《贺州市发展总体规划》，统领我市发展全局。健全规划衔接协调机制，增强规划的调控指引，突出全市发展总体规划的龙头地位，为城乡管理、土地利用、环保生态、交通建设、民生事业等专项规划编制提供依据和接口。同时，精减专项规划数量，增强专项规划编制的针对性和有效性。

强化空间管控能力。将经济社会发展与优化空间布局融为一体，按照“生态空间山清水秀、生产空间集约高效、生活空间宜居适度”的发展要求，落实“生态立市、产业强市、特色兴市”的工作举措，坚决守住生态保护空间，合理布局产业发展空间，科学界定城市潜在空间，不断优化土地增量布局，盘活土地存量管理，实现国土空间集约、高效、可持续利用，保障人口、产业、资源和环境的协调发展。

实现高效管理模式。建设“多规合一”涉及的用地边界、空间信息、建设项目等多元化信息统一平台，实现“一张蓝图”的规划平台、审批流程、反馈机制和监督体系。以此，深化行政审批改革，进一步减少审批环节和压缩审批时限，实现“报得进、审得快、批得出”，促进城市的高效管理。

（二）狠抓开放合作，当好向东开放排头兵。深入实施“向东发展战略”，以建设“贵广高铁经济带先行区”为引领，加快打造 “广西对接东部和中部的重要门户和枢纽”。

以园区为载体有序承接产业转移。按照“一区多园”“园中园”模式，抓好广西碳酸钙千亿元产业示范基地西湾园区、旺高工业区平桂分园、新型建筑材料产业园、钨钛锡产业园、稀土产业园等特色园区建设。按照“双驱工程”实施要求，尽快完成粤桂县域经济产业合作示范区发展规划的编制、评审工作，完善广西碳酸钙千亿元产业示范基地、贺州华润循环经济产业示范区、旺高工业区、钟山工业园区等重点园区基础设施建设，尤其要确保重点园区年内实现30万平方米并力争完成60万平方米标准厂房建设，力争年内实现园区基础设施投资100亿元以上。更加注重招商选资，严把投资强度、产出率、生态环保等准入门槛，深化与产业龙头企业、央企强企的合作，完成产业项目投资200亿元以上，实现重点园区新增工业产值100亿元以上。同时，以重点园区为载体，利用泛珠三角区域经贸合作洽谈会、中国—东盟博览会、美丽广西香港行、桂台经贸合作论坛和贺州农展会等平台，拓展与东盟、粤港澳、台湾等地合作交流；完善招商项目跟踪服务、督促检查机制，提高招商成功率、合同项目履约率、资金到位率和开竣工率，力争全年招商引资项目到位资金280亿元以上；积极培育进出口经营主体，提升对外贸易水平，力争完成外贸进出口1.76亿元，增长10%。

打造区域性交通枢纽和物流节点城市。按照建设“桂粤湘区域性交通枢纽”的发展要求，抓好2014-2016年综合交通基础设施大会战，推进涵盖铁路、高速公路、机场、城市路网、水路航运、农村道路的44个重点项目建设，加强与珠三角1小时经济圈的交通对接。同时，围绕重点园区和重大产业布局，全面启动建设一批现代物流、科技服务、专业市场、软件与信息、生态休闲养生等领域的现代服务业聚集区。要通过争设南宁海关贺州办事处、创建公路口岸、发展“铁海江”联运等形式，努力打造桂粤湘边界“无水港”；加快把贺州旺高新材料国际交易中心创建成为自治区级现代物流集聚区，重点推进旺高·国际石材城会展中心、贺州碳酸钙产品展示长廊、广西碳酸钙千亿元产业示范基地物流园、平桂旺高物流商贸城、贵广铁路钟山西站站前物流园、贺州市快递物流园等项目建设；规划建设自治区级商贸物流专业市场，重点抓好贺州古柏特色产业综合交易集散区、贺州市平桂新区新型综合市场、富川有机果蔬综合产业园等项目，打造区域性物流节点城市。

联手做大高铁沿线特色旅游品牌。抓紧实施桂贺区域一体化发展规划，加强与沿线城市互动合作，积极宣传推广贺州生态旅游等特色品牌，共同开拓客源市场。要推进与桂林建立政府联席会议、部门对口协商等合作机制，对接桂林国际旅游胜地建设，打造桂贺一体化旅游圈，融入粤桂黔大旅游区建设。完成昭平县广西特色旅游名县创建和申报工作，力争富川瑶族自治县进入广西特色旅游名县创建县行列；完成黄姚古镇创5A申报工作，继续推进姑婆山景区创5A工作；加快建设姑婆山景区游客服务集散中心、南乡森林温泉度假村、秀水状元村等重点旅游项目，进一步凸显贺州自然生态、人文景观、长寿养生资源优势，提升服务业标准化水平，加快构建“快旅慢游”服务体系，努力打造 “华南生态休闲度假养生旅游目的地”。

（三）狠抓转型升级，壮大特色明显的支柱产业。坚持资源优势与产业特色相结合，培育独具优势的产业竞争力。

扎实推进创新驱动。全面实施创新驱动发展战略，围绕碳酸钙、新型建筑材料、稀土等产业关键技术建立科技攻关平台，重点推进与中科院过程所和电子所合作的贺州创新基地、中科院亚热带农业生态研究所贺州生态经济研发和循环农业试验基地、广西碳酸钙产业技术研发中心和广西碳酸钙质量检测中心、高新技术产业开发区、科技企业孵化器、农业科技园区等创新平台建设，加大园区高新企业培植力度，推动发展动力向创新驱动转变。深入实施“引智入贺”工程，通过聘请产业发展专家、争设院士工作站、专利技术入股等多种模式，提高产业转型、经济发展的科技创新水平。

构建以“两个千亿元产业”为龙头的现代工业体系。抓住平桂石材资源综合利用示范基地列入国家资源综合利用“双百工程”的契机，重点建设中国冶金地质总局石材资源综合开发项目，打造布局合理、关联度高、互补性强的碳酸钙千亿元产业集群；强力推进广西旭腾实业集团新型建筑材料纳米科技板项目，加快发展以节能、环保、综合利用为重点的新型建材，以及资源加工废弃物为原料的新型墙体材料，打造新型建筑材料千亿元产业。同时，继续大力发展机械、电子信息、食品药品等战略性新兴产业。着重抓好日轻桂银偏析法高纯铝、桂航精密铸锻件、平桂飞碟钨系列产品深加工技改、华润啤酒二期工程、富川年产30万吨天然饮用水、华泰药业医药产业园等项目建设，打造“雁阵齐飞”产业聚集发展模式，年内新增规上企业30家以上。

提升特色农业发展质量。坚守全市16.77万公顷耕地保有量和14.41万公顷基本农田保护红线，粮食播种面积稳定在206万亩、总产量72万吨以上。完善农村基本经营制度，抓好农村土地承包经营权确权登记颁证工作，推进所有权、承包权和经营权三权分置，规范农村土地承包经营权流转市场规则，实施“小块并大块”土地整治10000亩以上。实施“新型职业农民培育”工程，发展种养大户、家庭农场、农民合作社和农业龙头企业等新型农业经营主体50个以上。强化农业企业清洁化标准化生产，完成10家企业产品“三品一标”认证，创建“广西农业标准化示范市”。加强自治区级农业科技园区建设，抓好五个县（区、管理区）自治区级现代特色农业（核心）示范区建设，培育和申报国家级农业科技园区。抓好农产品质量安全，争创“国家出口食品农产品质量安全示范市”。对接供港蔬菜“直通车”项目，扩大出口备案基地建设，提升发展“粤港澳果蔬基地”。

（四）狠抓生态经济，打造和谐友好的“生态贺州”。积极推动绿色发展、循环发展、低碳发展，精心呵护“山清水秀生态美”的特色品牌。

大力发展生态健康产业。尽快完成生态新城总体规划的编制、评审工作，抓好电子科技生态产业园的自治区级产城融合试点工作，落实《贺州市生态健康产业发展规划（2014-2025年）》要求，打造“生态健康旅游、生态健康休闲、生态健康养生和生态健康晚年”新型业态和特色品牌。规划建设自治区级健康养生集聚区，推进贺州市生态健康产业示范基地、生态昭平休闲养生示范区、贺州姑婆山国际养生度假区等工程项目，加快打造自治区级健康养生集聚区。同时，支持富川瑶族自治县创建国家循环经济示范县；以贺州华润循环经济产业示范区为核心，打造一批循环经济示范企业和清洁生产示范企业。

构建“生态宜居”的特色城镇体系。深入实施总投资150亿元的市容市貌综合提质三年行动，完善中心城区基础设施，重点抓好生态新城核心示范工程、“一江两岸”建设开发工程等八大工程，推进火车站综合交通客运枢纽工程、大钟山公园、八步文化旅游新城等项目建设，新铺设狮子岗排洪河截污工程、黄安寺排洪河截污工程等城区雨污分流管网100公里，城市建成区面积扩大至40平方公里。完善并落实市容环境综合整治长效机制，加强城区道路交通秩序管理，整治违法乱建、乱摆乱卖等突出问题，努力改善百姓的工作生活环境。实施好大县城战略，加快把昭平县打造成为独具特色的山水养生休闲度假区，把钟山县城培育成为广西中小城市，把富川瑶族自治县建设成为具有浓郁民族特色的“中国瑶乡明珠”。突出抓好全市17个重点镇建设，全面推进重点镇行政管理等八个方面的配套体制改革，实现“重心下移、权力下放”；抓紧全市16个旅游名镇创建、城乡风貌改造、特色文化名镇名村和传统村落保护利用工作，完善城镇供水、排污、用电、路网、天然气等基础设施。推进“美丽贺州·生态乡村”建设，健全清洁城镇乡村机制，抓好“村屯绿化”“饮水净化”“道路硬化”三个专项活动，重点打造25个市级精品示范村屯，培育形成功能互补、共建共享、风情浓郁的特色城镇带。

深入开展环境保护和治理。继续开展环境风险安全隐患大清查大整治行动。突出治理207国道平桂段扬尘污染。抓好规模化畜禽养殖污染治理。深入实施“绿满八桂”工程，完成造林21.3万亩，义务植树640万株。以龟石水库国家湿地公园项目建设和西岭山国家森林公园申报建设为抓手，强化贺州水源地利用和保护。推进天然林、水源林保护、中小河流域治理等重大生态工程。与相邻的永州、肇庆和清远等市加强环境应急区域合作，联手打造珠江中上游国家重要的生态安全屏障。重点抓好贺江、桂江等流域污染防治，推行河道管理“河长制”，提高环境监管和应急水平，确保“满江清水向东流”。

努力完成节能减排任务。积极抓好应对气候变化及节能减排工作，继续开展重点节能改造、公共机构节能改造和节能产品惠民工程。按期淘汰“黄标车”，减少机动车氮氧化物排放。全面执行污染物总量控制和排污许可证制度，强化建设项目环境保护“三同时”监管。按照“退城入园”的要求，对不符合城市总规、土地利用规划要求和环境污染问题突出的企业，加快组织实施搬迁、整改工作。

（五）狠抓项目建设，夯实经济社会发展的根基。在全市开展“项目建设年”活动，继续加大项目前期工作力度，谋划推进一批重大项目建设，保持项目建设“开工一批、在建一批、投产一批”的良好态势。

铁路方面。力争完成洛湛铁路永州至玉林段电气化扩能改造项目预可行性研究报告，完成柳州经贺州至韶关铁路项目方案研究报告并争取列入国家铁路“十三五”规划，加快推进贺州至梧州城际铁路项目前期工作，配合做好信都火车站改建工程及其他配套设施建设规划工作，争取完成贺州火车站改扩建工程项目设计并申报中国铁路总公司。

公路方面。紧抓永贺高速公路建设，确保2016年底前竣工通车；加快贺州至来宾高速公路（贺州段）前期工作，力争年内开工；加强与梧州市、清远市对接，加快梧州至信都高速公路、二广高速连山至贺州支线前期工作。实现省道301线灵峰至信都、国道207线贺街至信都段维修工程开工；推进国道207线贺州至富川一级公路、昭黄二级路绕城线（含马滩大桥）、323线贺州至钟山公路、富裕至马江二级公路、三合至马江二级公路、大汤经南乡至广东福堂二级公路、八达东路（贺州大道至莲塘马尾河大桥段）等项目建设。抓好长湾绕贺街二级路、路花至里松二级路、钟山经龟石水库坝首接麦岭538国道项目前期工作。

机场和内河水运方面。争取尽快完成贺州支线机场的选址、预可研、可研等前期工作；加快推进贺州通用机场、警用和应急救援机场的前期工作，力争年内开工建设。加快推进贺江扩能、桂江航道工程前期工作，建设马江作业区、马江航道站，完成贺江八步至夏岛段航道疏浚整治工程。同时，积极谋划在西江主航道规划建设“贺州（梧州）飞地港口”。

能源保障方面。加快推进电源建设、电网改造、县县通天然气、垃圾焚烧发电等项目前期工作，大力发展风电清洁能源，推进富川长广、钟山两安、大桂山等风电场和富川长春、朝东、金子岭风电场220kV配套输电线路等项目建设。完成总投资7800万元的11个农村水电站增效扩容改造任务。

农林水利方面。实施贺州市20万亩优质稻标准化基地建设、嘉宝食品标准化种植基地扩建等一批重点现代农业产业项目。抓好富川、钟山高标准农田整县推进工作。推进八步区小型水利工程管理体制改革全国试点县和富川瑶族自治县农田水利设施产权制度改革全国试点县工作。完成八步、富川、钟山小农水重点县项目建设任务。确保望高至担杆岭水厂原水输水管道建设和担杆岭水厂二期工程及城区供水管网建设工程等项目竣工。稳步实施路花水库工程（贺州市备用水源）和中小型病险水库除险加固工程，推进贺江、桂江等9条中小河流治理项目。实施龟石水库东干渠输水管道明渠改暗管工程（龟石至旺高段）项目建设。

（六）狠抓民生改善和社会事业，提升群众幸福指数。推进基本公共服务均等化，使发展成果进一步惠及全体人民。

强化就业和社会保障。认真落实鼓励支持就业创业的各项政策，重点抓好高校毕业生、返乡农民工等群体的就业创业工作。全市城镇新增就业1.2万人，城镇登记失业率控制在4.2%以内。加快推进金保工程“五险合一”信息系统建设，确保社会保险基金安全运行。有序推进农业转移人口市民化，努力实现城镇基本公共服务常住人口全覆盖。完善以城乡低保为核心的社会救助保障体系，构建以乡镇（街道）为责任主体的“一门受理、协同办理”社会救助综合服务窗口。以社会保险扩面征缴为重点，加快社会保险由 “制度覆盖”向“人群覆盖”转变。

坚持教育优先发展战略。深入实施教育振兴行动计划和八大重点工程，完成新建13所学前教育、7所义务教育学校的任务，抓好农村薄弱学校改造工程。完成自治区下达的九年义务教育巩固率、高中阶段毛入学率、学前三年毛入园率目标任务。发挥贺州职业学院辐射作用，深化职业教育综合改革。推动将贺州学院建成特色鲜明的应用技术型大学。抓好贺州市示范性综合实践基地、市职教中心教师公寓楼、贺州高中教师公寓楼、平桂高中迁建工程等教育重点工程建设。

加快发展卫计文体事业。继续推进县级公立医院改革，加强基层医疗、重大疾病防治等卫生计生服务体系建设。稳步推进城乡居民大病保险工作，确保城镇职工医疗保险、城镇居民医疗保险、新型农村合作医疗参保率稳定在95%以上。抓好卫生计生基础设施建设，全面深化诚信计生，加大奖励扶助、依法行政和优质服务力度，提高卫生计生治理能力。继续深入实施广播电视村村通、文化信息资源共享、农家书屋等文化惠民工程，加强市文化中心、文化“三馆”和村级公共服务中心建设，进一步完善公共文化服务体系。大力发展体育事业，积极备战广西第十三届运动会，提前做好2016年广西第八届农运会承办工作。

扎实推进精准扶贫。深入实施贺州市革命老区扶贫开发攻坚“三年帮扶行动”，争设南岭山区国家集中连片特困地区。完成“十二五”规划170个贫困村整村推进扶贫开发任务，年内实现减贫人口6.3万人，力争贫困地区农民人均纯收入达5100元以上。

强化社会治理创新。推进公安“八区”建设，完善社会治安防控体系。统筹推进新型农村社区建设，促进基础设施向农村延伸，公共服务向农村覆盖。治理餐桌上的污染，加快推进食品药品检验检测中心项目建设。畅通诉求表达渠道，维护群众合法权益。加强对安全生产、公共卫生、环境安全事件的预防预警、督促检查和应急处置。加强国防动员、国防教育和民兵预备役工作，争创全国双拥模范城，进一步密切军民关系。

实施年度重点民生项目。按照“突出重点，量化直观”的原则，抓好六方面重点民生项目，确保建成一个、满意一个、惠及一片。1.农村道路建设项目。完成103公里的6个县乡道联网沥青（水泥）路工程，83.2公里的29个建制村水泥（沥青）路工程。2.农村安全饮水工程。筹集资金9000万元，推进农村安全饮水工程建设，解决15万农村人口饮水不安全问题。3.农户住房保险工程。以政府出资参保与农民自愿参保相结合方式，筹集410万元资金为符合条件的农村住房购买政策性保险。4.城镇保障性住房项目。推进三期棚户区改造项目建设。开工建设公共租赁住房4646套，各类棚户区改造2.1万套。5.新建幼儿园项目。新建公办中心幼儿园13所，缓解“入园难”问题。6.绿化示范村屯项目。在2015-2016年计划投资1亿元左右，实施村屯绿化490个，绿化面积3000亩，种植树木20万株。

各位代表，搞改革、谋发展都是为了让人民过上幸福生活，我们要抓住人民最关心、最直接、最现实的利益问题，一件接着一件办，一年接着一年干，一起努力把“福”字写得更大、更好！

（七）狠抓体制机制改革，激发市场和社会活力。围绕解决发展面临的突出问题，推进既符合当前发展需要、又有利于长远制度安排的改革举措，促进经济提质增效、平稳增长。

深化关键领域改革。设立工业发展基金，扶持企业做大做强做优。大力发展混合所有制经济，完善国企投资公益项目的代建和回购制度，推动国有资本向优势产业和特色产业集中。完善工业园区体制机制，落实重点园区财政收入分配办法，提高园区集中财力办大事的调控能力；对园区内的项目建设试行全程协办、代办制度。争取自治区同意将县级电力公司纳入计划实施的峰谷分时电价方案；推进园区企业直购电试点工作，加快实现园区直供电，全力降低园区用电成本。

深入实行简政放权。做好行政审批事项“接、放、管”工作，全面实施工商登记“先照后证”。按自治区要求，全面放宽社会投资准入，支持民间资本进入市政公用、社会事业、金融服务等领域。通过创业引导基金、优惠贷款、技术市场等方式，推进非公经济规模化、特色化、专业化、高新化发展。

抓好财政金融改革。整合规范专项资金和各项财税优惠政策，严厉打击偷税漏税行为。做好地方政府性债务清理和甄别工作，建立规范合理的地方政府性债务管理及风险预警机制。积极推进“营改增”和消费税等税收制度改革。全面深化农村金融改革，探索农村产权交易市场建设。推进中小企业保证保险试点工作，使用好中小企业信贷补偿资金，努力缓解企业 “融资难”“融资贵”问题，力争新增贷款余额36亿元。

增强土地保障能力。严格按照中央、自治区的时间节点要求，做好用地报批和供地保障工作，确保市、县（区、管理区）近五年已批用地供地率达60%以上，使土地利用更加集约高效。通过向上争取新增建设用地指标、盘活存量和实施区位调整、低丘缓坡、增减挂钩等土地政策，保障重大项目、重点民生项目用地需求。加强补充耕地工作，特别是补充水田和旱地提质工作，切实做到“占一补一、占优补优、占水田补水田”，实现耕地占补平衡。同时，要研究完善项目用地征拆办法，切实保护好被征拆户合法权益，妥善解决历史遗留问题，依法依规加快推进征拆工作。

三、深入推进依法行政，加快建设法治政府

按照自治区政府的部署，遵循“职能科学、权责法定、执法严明、公开公正、廉洁高效、守法诚信”要求，推进依法行政，建设法治政府，保证人民赋予的权力始终为人民谋利益。

（一）依法推进政府职能转变。坚持“法定职责必须为，法无授权不可为”，最大限度减少对微观事务的干预。制定公布权力清单、责任清单、负面清单和财政专项资金管理清单。严格遵守中央“八项规定”、自治区“两规定一办法”、厉行节约反对浪费条例、公务接待管理规定等各项政策规定，坚决纠正“四风”。实行“三公”经费、会议费、培训费等一般性支出“零增长”。按自治区工作部署，完成全市公务用车改革。

（二）严格依法行政。建立健全依法决策制度，坚持把公众参与、专家论证、风险评估、合法性审查、集体讨论决定作为重大决策的必经程序，确保决策制度科学、程序正当、过程公开、责任明确。加强行政机关内部重大决策合法性审查和风险评估、执行效果评估。深化行政执法体制改革，推进综合执法，着力解决权责交叉、多头执法问题。严格行政执法人员资格管理，加强行政执法监督，切实规范执法行为。

（三）加强权力制约和监督。加强审计监督，对公共资金、国有资产、国有资源和领导干部履行经济责任情况实行审计全覆盖。自觉接受人大的法律监督和政协的民主监督，坚持政府重大决策出台前向人大报告。加强民主协商，认真听取各民主党派、工商联、各人民团体和社会各界人士意见。及时办理人大代表的议案、建议和政协委员的提案。充分发挥监察部门专门监督的作用，高度重视人民群众监督和新闻舆论监督。

（四）加强经济调节和市场监管。建立有效的监管网络和科学的监管机制，严厉惩处污染环境、危害食品药品安全、假冒伪劣、侵犯知识产权、违背市场公平竞争原则的行为。建立科学的抽查制度、责任追溯制度、经营异常名录和黑名单制度，重点监控问题企业和违法违规经营者。加强中介组织培育和规范，完善诚信体系和评估体系建设。

（五）全面提升行政效能。推进政务公开规范化、信息化，加强互联网政务信息数据服务平台建设，强化政府网站信息发布工作，加大重点领域和关键环节的政府信息公开力度。全面推进预算绩效管理工作，大力推广政府购买服务。开工建设新的市级政务服务中心，完善各县（区、管理区）政务服务中心，实现全市100%的村（社区）建成政务服务中心。巩固“不作为、乱作为”专项整治活动成效，对政府工作报告主要任务、重大项目进展和重点事项落实情况，以台账式管理进行督促检查，构建更严格有效的目标管理责任机制。

各位代表，贺州正处在改革攻坚、爬坡过坎的关键时期，机遇与挑战并存，做好今年经济社会发展各项工作，使命光荣，责任重大。让我们在市委的领导下，咬定发展不放松，面对困难不退缩，尽职尽责，扎实工作，为加快打造贺州经济社会发展“升级版”而努力奋斗！
